# Piezoelectric needle sensor reveals mechanical heterogeneity in human thyroid tissue lesions

**DOI:** 10.1038/s41598-019-45730-x

**Published:** 2019-06-26

**Authors:** Shivani Sharma, Renato Aguilera, JianYu Rao, James K. Gimzewski

**Affiliations:** 1 Department of Pathology and Laboratory Medicine, David Geffen School of Medicine at University of California, Los Angeles (UCLA), Los Angeles, CA USA; 2 California NanoSystems Institute, UCLA, Los Angeles, CA USA; 3 Jonsson Comprehensive Cancer Center, UCLA, Los Angeles, CA USA; 4 Clinical and Translational Science Institute, UCLA, Los Angeles, CA USA; 5 Department of Chemistry and Biochemistry, UCLA, Los Angeles, CA USA

**Keywords:** Diagnostic markers, Thyroid cancer

## Abstract

Palpable thyroid lesions are common, and although mostly benign, lethal malignant nodules do occur and may be difficult to differentiate. Here, we introduce the use of a piezoelectric system called Smart-touch fine needle (or STFN) mounted directly onto conventional biopsy needles, to evaluate abnormal tissues, through quantitative real-time measurements of variations in tissue stiffness as the needle penetrates tissue. Using well-characterized biomaterials of known stiffness and explanted animal tissue models, we first established experimental protocols for STFN measures on biological tissues, as well as optimized device design for high signal-to-noise ratio. Freshly excised patient thyroids with varying fibrotic and malignant potential revealed discrete variations in STFN based tissue stiffness/stiffness heterogeneity and correlated well with final histopathology. Our piezoelectric needle sensor reveals mechanical heterogeneity in thyroid tissue lesions and provides a foundation for the design of hand-held tools for the rapid, mechano-profiling of malignant lesions *in vivo* while performing fine needle aspiration (FNA).

## Introduction

Evaluation of a thyroid nodule remains one the most common and yet one of the most challenging problems for endocrinologists. Thyroid nodules are common with ~1% of males and 5% of females having palpable thyroid nodules^1^. Among these, a majority (>90%) of the thyroid nodules are non-malignant or well-differentiated papillary and follicular carcinomas^2^. Nevertheless, the remaining ~10% of cases may cause significant morbidity and eventually death. The standard diagnostic modality of evaluating malignancy in thyroid nodules involves ultrasound coupled with fine needle aspiration cytology (FNAC) primarily to prevent unnecessary surgeries for benign conditions or avoid missing malignant nodules^3^. However, the overall false negative rates (malignant histology of a nodule with benign cytology) for FNAC ranging from <1 to 12%^4,5^, and indeterminate cytological findings, warrant the need for new methodologies to improve diagnostic sensitivity and accuracy of thyroid FNAC.

Increased tissue stiffness is a widely accepted and actively studied biomechanical property of fibrotic tumors and has been linked to several hallmarks of cancer^6^, including growth, metabolism, invasion, and metastasis^6–8^. Biomechanically, thyroid lesions exhibit inhomogeneous elastic behavior as a consequence of collagen in the stroma and frequent calcium deposits^9^. Such regions vary by orders of magnitude in stiffness behavior from healthy cells^10^, and hence vary in haptic feel. However, rapid and quantitative methods for measuring tissue stiffness that can be translated into clinical settings have not yet been established. Elastography has yielded promising results in the diagnosis of breast^11–15^, liver^16^ and pancreatic lesions^17,18^ though histological confirmation remains the treatment standard.

Needle-based sensors provide greater access to specific bio-component contributions in tissue mechanics^19^ and bridge the gap between cellular^20–22^ and tissue level measurements at high spatial resolution (e.g., different tissue planes or variations of densities within the tissues). Needle-tissue interactions have been previously attempted to guide tumor navigation, but it is difficult and expensive to micro-fabricate the piezo-sensor (<100 μm) used inside a hollow needle^19^. The Hansma group used a reference probe technique to measure mechanical properties of normal and diseased soft and hard tissues *in vivo* and *in vitro*^23^. However, the technique, mostly employed to measure bone material properties had a limited measurement range (<600 μm) and the spatial resolution in the mm scale (0.2–2 mm). A combined micronewton-resolution, tensiometric force probe, and micromanipulator were used to measure the variation in tissue stiffness (100 µm to mm scale)^24^. However, the thickness of the samples that can be accurately measured significantly limits applicability. Previous studies probed the nature of FNA needle penetration during thyroid FNA biopsy for cancer biomarkers^25,26^ but yielded only qualitative tactile assessment of nodule stiffness using their fingers resulting in operator bias, and lack of quality control. Thus, there remains an urgent and unmet need for new and quantitative technologies to detect and profile solid tumors such as thyroid lesions, based on their biomechanical characteristics.

Here, we introduce the use of a simplified piezoelectric needle sensor to measure thyroid tissue stiffness at unprecedented cellular scale resolutions. To the best of our knowledge, this is the first quantitative biomechanical report on stiffness heterogeneity of thyroid lesions. Unlike, other commonly used single cell-based biomechanical techniques such as the micropipette aspiration^27^ and atomic force microscopy^22,28–32^, our ‘smart-touch fine needle’ (or STFN) is a low-cost method that enables quantitative real-time biomechanical analysis of thyroid tissues within the cellular and stroma microenvironment. The technology is about ten times finer than the diffraction limit of Ultrasound, Magnetic Resonance Imaging or Computer Tomography, bridging size scales from the cell, tissue and organ level to reveal distinct nano-mechanics of thyroid tissues from different histo-pathologies. Coupled directly onto the conventional fine biopsy needle (25 Gauge), the smart-touch can be easily adapted to biomechanically evaluate thyroid lesions for risk of malignancy or aggressive cancers and has the potential for use in clinics in outpatient point-of-care settings in future.

Previously, we demonstrated the ability of a piezoelectric force-sensing needle to differentiate solid and fluid-filled thyroid nodules^33^ biomechanically. Significantly higher force variations (1-D force heterogeneity and stiffness heterogeneity) were noted in solid nodules compared to fluid nodules or regions corresponding to healthy thyroid tissue. However, these observations were limited to ultrasound neck phantom observations. Employing next-generation needle sensor design in the current study, we introduce the prototype design for STFN mounted directly onto conventional needles used during FNAC. Using data obtained via STFN prototype, we show that thyroid lesions with varying fibrotic and malignant potential reveal discrete variations in tissue stiffness/stiffness heterogeneity, and correlate well with final histopathology data. Based on our findings, we believe that STFN provides a solid foundation for the development of sensitive and low-cost tools that enable rapid, quantitative, and radiation-free stiffness heterogeneity-based characterization of malignant lesions *in vivo*, in thyroid and which applies to other solid tumors.

## Prototype Development

**Figure 1:**
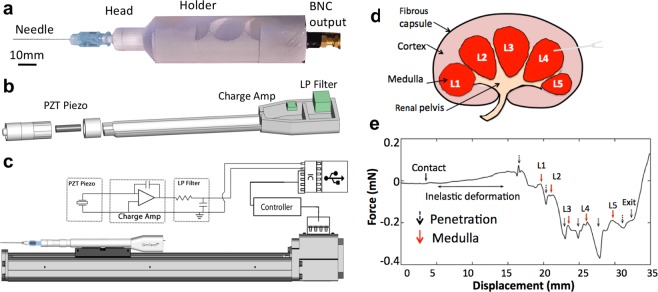
Experimental set-up for the smart-touch fine needle (STFN). (**a**) Optical image of the STFN device composed of a 25G fine needle, PLA polymer housing and connected to RG-58/U coaxial BNC cable. (**b**) Design schematic of housing attaches the needle to piezoelectric tube transducer and piezo-response measured through 20 AWG twisted pair Cu wire. The twisted pair is frayed to connect to the BNC. (**c**) An illustration shows the experimental connection diagram of STFN. (**d**) Schematic diagram of a cross-section of porcine kidney samples showing fibrous capsule, cortex and medulla regions. (**e**) An example of force versus needle displacement profile from STFN penetrating through the tubules of the kidney sample. Before the estimated point of contact (marked with a solid black arrow) between the needle and kidney tissue, the force observed was minimal. After the point of contact (solid black arrow), there is an elastic deformation due to fibrous capsule until an abrupt Hertzian penetration (broken black arrow) occurs. Subsequent deformation peaks occur (labeled L1 to 5) before full penetration at the point marked as exit (broken black arrow).

**Figure 2:**
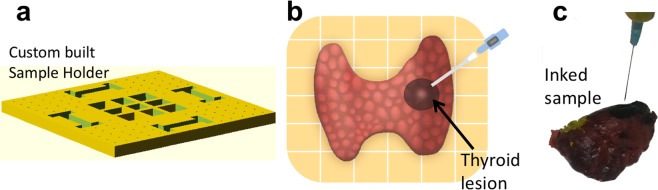
Experimental workflow during the typical STFN measurements and distribution of tissue stiffness heterogeneity observed for thyroid carcinoma and the healthy thyroid. Following a standard operating procedure, samples were first prepared by attachment and orientation into quadrants, using (**a**) biocompatible sample holders with calibrated grids as illustrated schematically in (**b**). (**c**) An inked patient sample measured using STFN. Samples were later processed for standard tissue histology.

The STFN device was constructed using additive manufacturing technology (3D printing) to allow for inexpensive and flexible exploratory design. A lead zirconate titanate (PZT) ceramic piezo cylinder (0.125″ (OD), 0.085″ (ID), and 0.500″ length) from Boston Piezo-Optics, Inc. served as a force transducer for measuring biomechanical properties. An ergonomic custom CAD designed (SolidWorks 2013) housing unit is prepared using a MakerBot 2 3D printer (Fig. 1a–c). The housing design incorporated several cutouts sized for each device element with the appropriate dimensions. Double twined 25 AWG Lakeshore Teflon insulated copper wires were attached using conductive silver epoxy for all connections and electrically isolated throughout the holder. The printed parts were assembled using fast drying cyanoacrylate. Standard Becton Dickinson (BD) 25 Gauge 3.50″ fine needle was attached to the piezo via a Luer lock as a measurement probe. Vibrational isolation was achieved by using a freestanding apparatus where all mechanical contact was pneumatically damped using an inexpensive custom vibrational liquid filter. Vibrational damping through liquid media produces a 3 dB filter within the low-frequency band <200 Hz [Fig. S2]. Measurement automation was controlled mechanically using a single axis Misumi LX26 actuator, where contact between the STFN device and actuator was damped using our vibrational filter. The experimental setup used a custom biocompatible sample holder alongside a compact analysis platform (as shown in Fig. 2a).

## Data Acquisition/Format

Measurements were taken using National Instruments USB-6259 data acquisition module routed from a Stanford Research Systems Low-Noise Current Amplifier. The mechanically induced electric current from the PZT piezo was converted to a voltage signal via a charge amplifier and its time trace recorded. The force trace was calculated in real-time through standard d_31_ Piezo equation [Eq. 1]. Needle actuation was controlled through using a linear motion actuator. We calculated the position trace of the needle from the Musimi LX26 motor’s known stepper size and rate. The optimum actuation speed was calibrated to 8 mm/sec to emulate clinical operation. Since needle and sample deformations during the operation were minimal, the force trace in combination with the position trace was converted from F(t) to F(x). All data were stored in an ASCII format and analyzed using LabView 2015 software.

## Quantitative Control Experiment of ex vivo Tissues

To better recapitulate the native tissue microenvironments within thyroid samples, here, we chose first to calibrate the device while including the effects of biological environments via an *ex vivo* animal tissue model. A control experiment using porcine kidney samples aided to develop a working model of spatial variations versus biomechanical variations in different optically identifiable regimes. We obtained a preliminary characterization of the axial forces that arise during needle insertion into a freshly excised porcine kidney, in terms of needle-tissue penetration, insertion peak force and apparent stiffness, and to attribute observed force peaks to specific tissue structure components.

Excised kidney organs were procured from traditional vendors and prepared using the standard procedures highlighted in the Standard Operation section while omitting any clinical reference. Figure 1d shows a schematic overview of the kidney, where biologically relevant features are labeled. A simplified structure of the organ comprises of medulla lobes (1–2 mm), various blood vessels (0.5–1 mm), and connective tissues within the renal region (>5 mm). Automation and experimental parameters for needle-tissue interactions were chosen to measure the biomechanical characteristics of the medulla and blood vessels due to their similarity in scale to typical human tumor nodules. Nominal parameters of needle speed and needle type (diameter expressed in wire gauge G; length, and shape) were chosen to simulate clinical methods typical for standard fine needle biopsy of thyroid lesions and to minimize tissue displacement^34^.

In a typical force-displacement profile (Fig. 1e), an initial approach of the needle towards the sample is characterized by a zero load force on the needle traversing through the free air before the estimated point of contact. The region following contact (I), exhibits positive force on the needle with initial elastic deformation (I, II) as modeled by the Hertzian formulation^35^. Later, a penetrative peak (broken arrow, Fig. 1e) was observed with characteristic spring-like compression and decompression force loads (II, III), shown as a relatively negative peak followed by a relatively positive peak. The spring-like cortex region between penetration and L1 show uniform negative (repulsive) response, except for the transient response due to spring relaxation. Subsequent peaks labeled L1-L5 were positive and attractive areas presumably corresponding to the vesicle-like structure of the medulla. The negative regions between peaks likely corresponded to hard repulsive tissue such as the major and minor calyx. An atypical hard region between L4-L5 corresponded to a region of calcified vessels verified visually in a subsequent dissection [Fig. S3] of the specimen. We estimated the dimensions of the medulla lobe by calculating the width from the full-width-half-maximum of each peak labeled L1-L5. The number of force peaks depends on the tissue structures that are encountered. Visual inspection of the sample reveals the width of the peaks to be ~2 mm which corroborate with the predicted literature value dimensions for kidney tubules. Secondary peaks found in each lobe were attributed to connecting blood vessels and similarly analyzed. The calculated width was ~1.87 mm and ~0.53 mm for the medulla lobe and blood vessels respectively. Based on anatomical considerations (Fig. 1d), results obtained from explanted samples of the kidney in a non-diseased state, accurately predicted the expected kidney architecture and biomechanical properties of the different regions, corroborating with literature values within <5%^36^. The developed analytical model for classifying biomechanical responses based on the identification of characteristic points on the needle force-displacement curves was employed for subsequent *ex vivo* patient thyroid sample analysis.

## Methods

### Protocol development and optimization of STFN measurements of *ex vivo* patient thyroid specimens

#### *Ex vivo* thyroid sample procurement

We followed a standard procedural protocol for every attained thyroid sample and measurement. Explanted human thyroids samples were procured from the Ronald Reagan UCLA Medical Pathology Department in accordance with the IRB protocol #**17-000418**. All methods were carried out in accordance with UCLA IRB guidelines and regulations. The UCLA IRB approved all experimental protocols. Since the tissue specimens were de-identified, the informed consent requirement was waived. The study was determined to be exempt from the UCLA JCCC ISPRC committee scientific peer review based on the following reason: the study involves the use of Tissues (i.e., from an existing tissue bank, creating a tissue bank, genetic epidemiology, and collection of tissues for lab based trials (such as biomarker research) that pose no more than minimal risk to subjects).

#### Sample mounting

First, the possible areas of interests for biomechanical profiling with the STFN were identified, in consultation with the pathologists, for each specimen studied. The initial diagnosis and relevant medical data were kept in a blinded secured database for reference. Measurements were taken within one hour after surgical removal while held in an environmentally controlled storage unit at 4 C to prevent sample degradation as reported previously^29^. Typically, thyroid samples were then placed on our custom sample holder (Fig. 2a) where the sample was mapped into topographic sections using the holder and a mesh grid. Assigned reference ID and a digitally scanned image for each sample were recorded in a secured database for future reference. The sample holder with the thyroid specimen was then placed in a normal position with the path of the STFN measurement apparatus, allowing for approximately 1 cm of free translation before contact of the needle with the sample. The initial 1 cm data was used for zero-force calibration and determining signal-to-noise ratio.

Careful attention was taken to ensure the correlation of STFN measurement with corresponding histology. For sample orientation, precisely oriented maps were prepared for each specimen to indicate the area(s) investigated for biomechanical analysis (Fig. 2b). We used color ink on the specimen (Fig. 2c) to highlight the specific area(s) that have been measured/sampled, as follows: Green (Area 1) - Nodule of primary interest (as defined clinically, the main factor for thyroidectomy); Red (Area 2) – Non-diseased area immediately adjacent to the main nodule (area 1); Black (Area 3) – Non-diseased area at least 0.5 cm away from the main nodule (area 1); Yellow (Area 4) – Contralateral thyroid without disease; Purple (Area 5) – Other nodule, if present. No more than five nodules/areas were analyzed for each specimen. Tissue sections were taken from the corresponding areas that have been measured and indicated as such on the gross description. STFN data analysis was performed independently without knowledge of histological diagnoses.

#### Histology analysis

After STFN measurements, inked samples were formalin-fixed and paraffin-embedded according to standard histological procedures. The subsequent histo-pathological examination included assessing the type of lesion and usual histo-pathological features (extent of tumor infiltration, fibrosis, necrosis, and lymphocytic infiltration).

#### Identification of characteristic points on STFN based needle force-displacement curves

**Figure 3:**
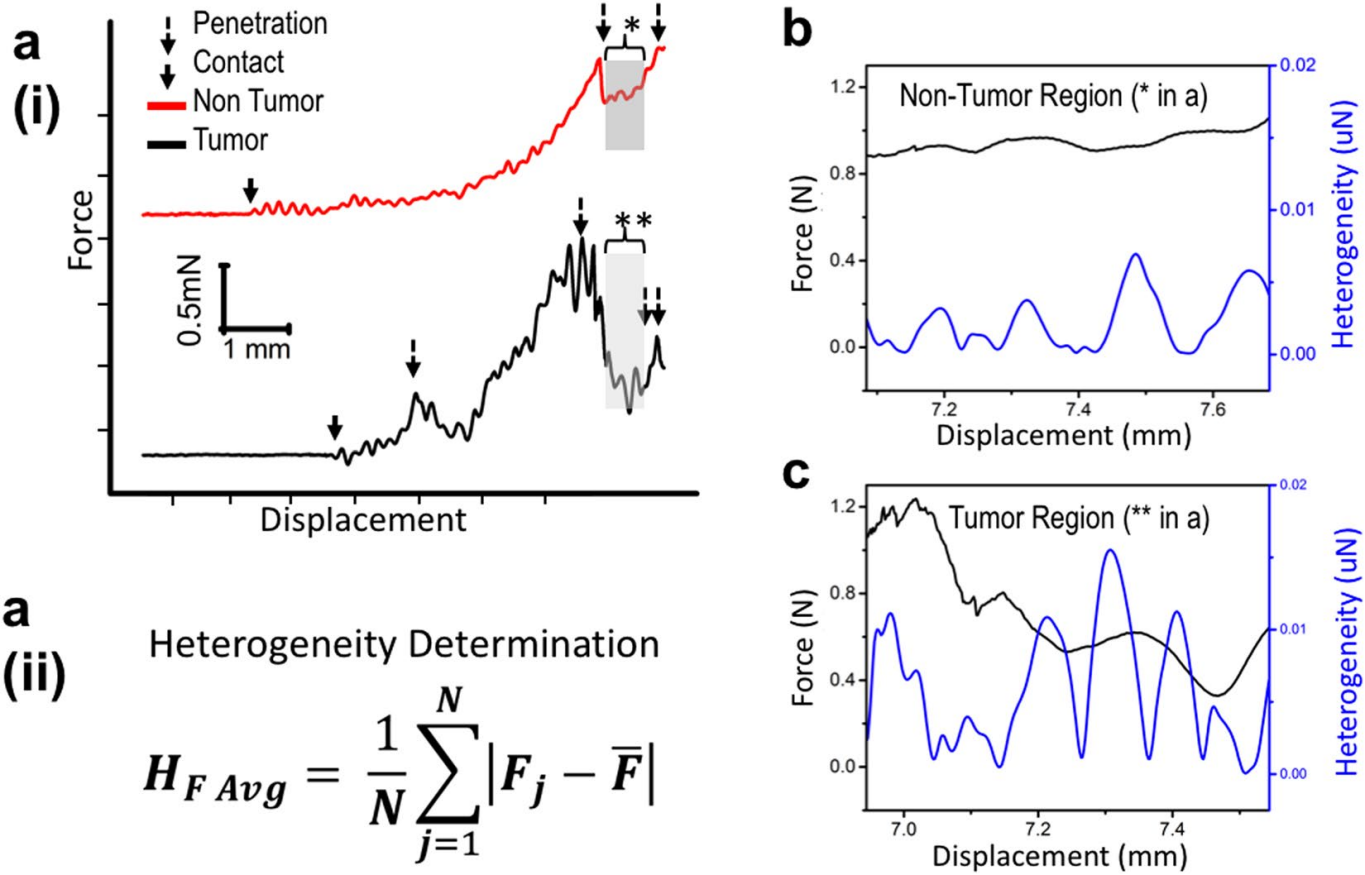
Method for determining the presence and location of the nodules in *ex vivo* human thyroid samples- based on needle biomechanical response. a.i, Shows the characteristic STFN response for the initial point of contact between the needle and the tissue sample (solid black arrows), followed by penetration (marked by broken arrows) into non-tumor and tumor tissues shown in red and black curves respectively. Malignant specimens (black) show several broken arrows corresponding to secondary interfaces caused by tumors as corroborated by histology. Within the identified regions of interest (marked with * and ** for non-tumor and tumor samples respectively), heterogeneity of tissue stiffness is analyzed based on Equation 1 given in **a(ii)**. (**b**,**c**) Show corresponding force-displacement curves from ROI for non-tumor (region marked with * in red curve in a.i), and tumor (region marked with ** in black curve in a.i) samples respectively. Representative *ex vivo* measurements of human thyroid following standardized operating procedures, show distinct responses between malignant and benign samples.

First, we measured the non-nodule regions of the thyroid tissue specimens as determined by haptic palpations. Real-time force versus needle displacement data from the STFN was observed and analyzed to determine the validity of each measurement (Fig. 3a). We developed standard direct-indicators of successful characterization through multiple heuristic trials. The Hertzian deformation response (Fig. 3a, red and black)[] indicated penetration of the needle into the tissue. Simultaneously the STFN response in stiffness deflection (Fig. 3a, red and black) characterized tissue granularity, indicating the presence of nodules or other extracellular material. Each measurement was repeated 5–10 times on different parts of the same nodule for statistical convergence. Subsequent STFN measurements targeted other areas of interests such as tumor nodules, cystic nodules, and lymph nodes following identical procedures. We inked each measured area for subsequent histological assessment of the thyroid tissue specimens. The order of the procedure was chosen to prevent region-to-region cross-contamination in histological studies.

### Human subjects approval, accordance and informed consent

Explanted human thyroids samples were procured from the Ronald Reagan UCLA Medical Pathology Department in accordance with the IRB protocol #17-000418. All methods were carried out in accordance with UCLA IRB guidelines and regulations. The UCLA IRB approved all experimental protocols. Since the tissue specimens were de-identified, the informed consent requirement was waived. The study was determined to be exempt from the UCLA JCCC ISPRC committee scientific peer review based on the following reason: the study involves the use of Tissues (i.e., from an existing tissue bank, creating a tissue bank, genetic epidemiology, and collection of tissues for lab based trials (such as biomarker research) that pose no more than minimal risk to subjects).

## Results

### Analysis of tissue stiffness and stiffness heterogeneity in patient thyroids

Using STFN prototype, we analyzed the tissue stiffness and stiffness heterogeneity of *ex vivo* patient thyroid samples including various thyroid histo-pathologies. Our goal was to quantify any significant differences in the biomechanical profiles for papillary carcinoma, papillary carcinoma with cystic change, tall-cell variant carcinoma (considered more aggressive), and carcinoma associated with Hashimoto thyroiditis, compared to non-tumor tissue including normal thyroid tissue and adenomatoid nodules with or without cystic components. Our findings provide the initial steps towards a mechanoprofile catalog of various types of carcinoma based on localized tissue biomechanics. Table 1 shows patient characteristics from all *ex vivo* thyroid samples analyzed in the current study. All measurements were done on site to simulate the clinical use of the device and reduce possible sources of contamination. Figure 3a shows typical force versus displacement profiles obtained using STFN.

**Table 1:** Statistical analysis of tumor variants. STFN data for 76 measurements are statistically analyzed according to their corresponding histology reports.

No.	Age/sex	Clinical History	Cytological/Histology
1	45/M	Papillary Thyroid Carcinoma 2.6 cm tumor	Positive for metastatic malignant cells
2	52/M	Papillary Thyroid Carcinoma 1.2 cm pT3 pN0	Positive for metastatic malignant cells
3	64/M	Cystic Papillary Thyroid Carcinoma 0.8 cm	Positive for metastatic malignant cells
4	47/F	Hyperthyroidism and Thyroid Goiter	Negative for malignancy, cyst lined follicular cells
5	73/M	Papillary Thyroid Carcinoma 1.7 cm pT3 N1b	Positive for metastatic malignant cells
6	29/F	Papillary Thyroid Carcinoma with Hashimoto thyroiditis 1.5 cm pT3 N1a	Positive for metastatic malignant cells
7	64/M	Cystic Thyroid Goiter with Gout	Negative for malignancy, multinodular goiter
8	23/F	Hyperparathyroidism post parathyroidectomy	Positive for metastatic malignant tall cells, papillary thyroid microcarcinoma pT3Nx
9	33/M	Papillary Thyroid Carcinoma 1.1 cm	Positive for metastatic malignant tall cells
10	68/M	Papillary Thyroid Carcinoma 4.5 cm	Positive for metastatic malignant cells
11	49/F	Papillary Thyroid Carcinoma 1.6 cm	Positive for metastatic malignant cells
12	69/M	Hashimoto thyroiditis 0.2 cm	Positive for metastatic malignant cells

Variants of benign samples (Adenomatoid and Normal) show similar qualities while malignant samples (others) show drastic variability. Classification of each variant can be observed through their heterogeneous response using Principal Component Analysis.

#### Determining the presence and location of the nodules in *ex vivo* thyroid- based on needle biomechanical response

Similar to our control experiment (Fig. 1e), the initial approach regions prior to estimated contact between the needle and the tissue show a near-zero loading force (Fig. 3a) whereas the contact regions show typical Hertzian behavior. The point of initial contact between the needle tip and the sample is marked with solid black arrow. As seen in Fig. 3a, the length of the Hertzian region inherently differs from sample-to-sample, due to variations in size of the thyroid samples. As seen in Fig. 3a, the length of the Hertzian region inherently differs from sample-to-sample, due to variations in size of the thyroid samples. However, precisely identified needle-tissue insertion points followed by high-resolution sampling of localized force-displacement data within the tissue region of interest, results in thyroid sample size independent analysis. The points of needle penetration (broken arrow, Fig. 3a) at differing interfaces exhibit spring-like behavior and high stiffness. In case of a representative benign sample (Fig. 3a, red), only a single penetration event is noted (broken arrow Fig. 3a), followed by translation into a relatively homogenous non-nodule region. In contrast, the representative malignant sample shows three such events (Fig. 3a, black). The initial penetration corresponds to the transition from non-contact to initial contact with the thyroid tissue (regions marked as I in Fig. 3.a.i), while the subsequent penetration peaks correspond to the interface from non-nodule to nodule and out of the nodule (regions marked as II, III in Fig. 3.a.i), as the needle traverses through the specimen. A qualitative analysis of the real-time force versus needle displacement profiles obtained via STFN readily distinguished between benign and malignant thyroids (Fig. 3a).

**Figure 4:**
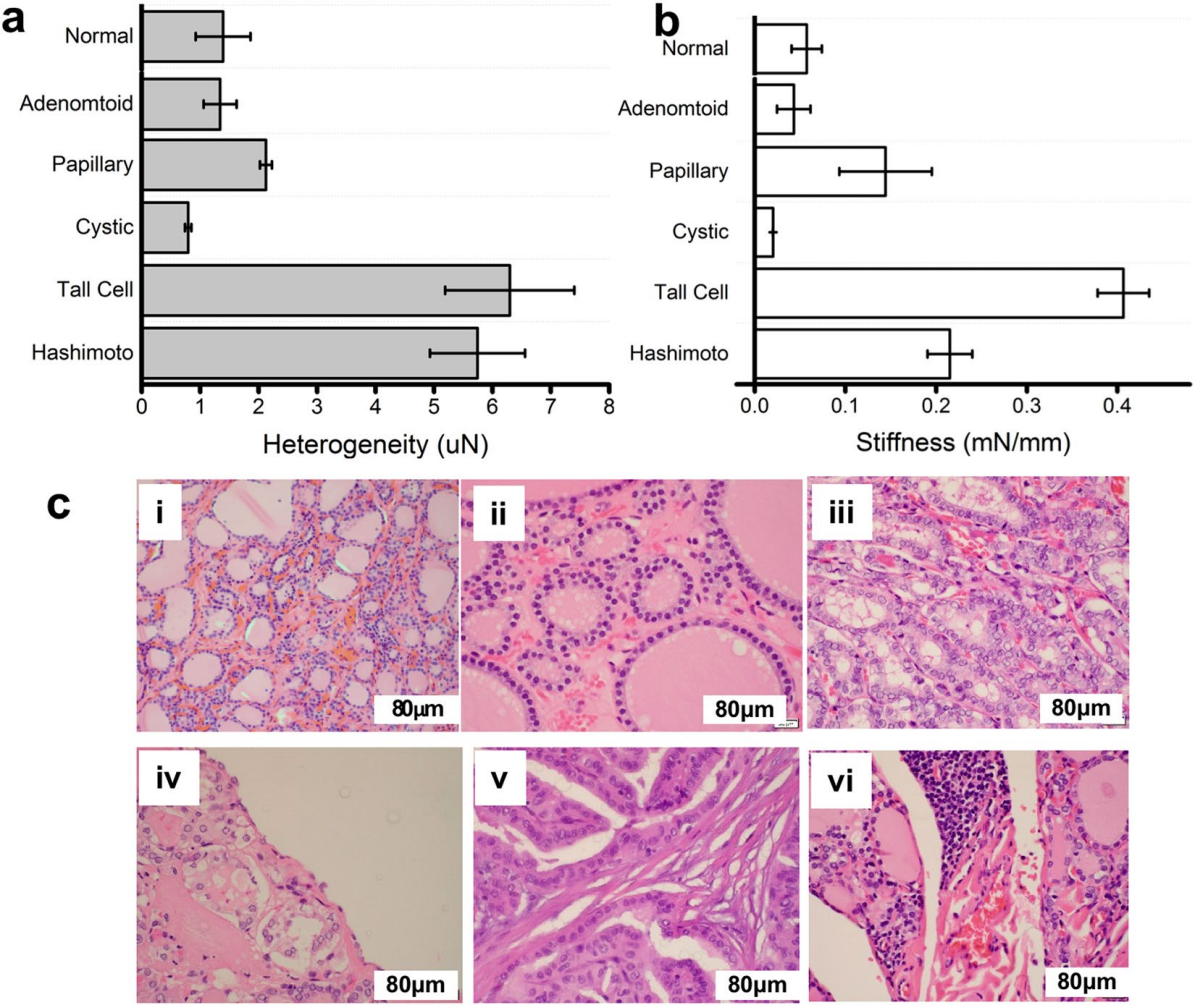
STFN based quantitative biomechanical analysis of patient thyroid tissue samples with corresponding representative histology. Corresponding data in (**a**,**b**), were obtained from one normal, one adenomatoid, four papillary, two cystic, three tall cell and two Hashimoto thyroiditis patients samples as described in Table 1. (**a**) Tissue stiffness heterogeneity (uM) and (**b**) tissue stiffness (mN/mm) evaluated for all thirteen patient samples studied. Data are stratified based on tissue variant types. Variants of benign thyroid samples (normal and adenomatoid) show similar biomechanical characteristics compared to malignant samples (others). (**c**) Histology data corresponding to each thyroid tissue variant represent (i) normal, (ii) adenomatoid, (iii) papillary of usual type, (iv) papillary carcinoma with cystic component, (v) tall cell variant, and (vi) Hashimoto disease respectively.

Needle-tissue penetration points were readily identifiable for all samples and were used to validate the presence or absence of nodules, as well as their positions. Further analysis of localized region-of-interest (ROI) determined the intrinsic biomechanical properties of malignant (Fig. 3a*; 3c) and benign (Fig. 3a**; 3b) human thyroids. Equally binned subsections of data from Fig. 3a are selected, representing non-tumor and tumor tissue labeled with solid arrows. Eq. 1 provides a quantitative description to calculate their force heterogeneity. For user validation, Eq. 1 is truncated to analyze 40 um windows and applied as a sliding function across the ROI in Fig. 3b–c. Our classification analysis uses the full 350 um range of the sectioned data to render quantitative insight presented later in Fig. 4.

For quantitative analysis of biomechanical variations between malignant and benign human thyroids, we measured stiffness heterogeneity as shown in Fig. 3b,c. The force heterogeneity profiles of the benign case show several areas of relatively high heterogeneity (illustrated in the representative profile shown in Fig. 3a, red). This characteristic was determined to be the typical composition of normal thyroid tissue due to normal variation in the density of hormone glands and blood vessels. In contrast, areas displaying about twice the amplitude in heterogeneity characterized the malignant case (in Fig. 3a, black). We attribute the increase in heterogeneity to the likelihood of tissue fibrosis and frequent calcifications in thyroid tissues, and which corroborate with histological analysis of the samples used in our study [Fig. S4]. Specifically, to examine differences between benign and malignant thyroid lesions, we used a non-parametric Wilcoxon rank sum test to compare the median tissue stiffness and stiffness heterogeneity differences measured between the two groups, with α = 0.05. Our results suggest large and statistically significant (P < 0.05) differences between median stiffness values obtained for benign (0.05 ± 0.02 mN/mm) versus malignant thyroid lesions (0.18 ± 0.15 mN/mm) (P = 1.55 × 10^−4^) in 12 patient nodules measured. Furthermore, stiffness heterogeneity was also found to be statistically higher in malignant compared to benign thyroid lesions (3.23 ± 2.49 μN; 1.41 ± 0.43 μN; P = 2.22 × 10^−5^).

#### Correlation between biomechanical measurement with histology at case-to-case level

Next, we analyzed STFN based stiffness and stiffness heterogeneity measured in several thyroid carcinomas with various histological features. A statistical summary of our findings is presented in Fig. 4(a,b) indicating stiffness heterogeneity (μN) and stiffness (mN/mm) respectively, evaluated for multiple non-nodule and nodule regions from all thirteen patient samples included in our study. These included the usual papillary carcinoma, papillary carcinoma with cystic change, tall-cell variant papillary carcinoma, papillary carcinoma associated with Hashimoto thyroiditis, and benign conditions including adenomatoid nodule (Table 1). Information on the specific variant cell-type was acquired using histological reports and cross-examined with the post-analysis results from STFN measurements.

Compared to normal thyroid tissue (1.39 ± 0.47 μN, 31 regions measured from patients with non-disease), adenomatoid samples showed low heterogeneity (1.34 ± 0.28 μN; n = 9). Cystic carcinomas were identifiable through characteristically low stiffness and heterogeneity (0.79 ± 0.05 μN; n = 5). In contrast, aggressive tall cell carcinoma showed significantly higher heterogeneity (6.29 ± 1.10 μN). A similar profile is shown for Hashimoto (5.75 ± 0.81 μN) syndrome in heterogeneity but can be differentiated using stiffness.

#### Correlation between thyroid histology and biomechanical characteristics at the tissue level

Corresponding structural variations within the different thyroid tissue histology were correlated with STFN biomechanical analysis for different thyroid tissue variants (normal, adenomatoid, papillary, cystic, tall cell or Hashimoto disease) are shown in Fig. 4c. Healthy thyroid tissue displayed follicular cells uniformly distributed with purple epithelial cells traversed within the collagen fibers (as represented in Fig. 4c-i**)**. Adenomatoid samples consisted of the same structure as a healthy thyroid except for follicular cells without nuclear features of papillary carcinoma forming uniform follicle with the modular formation (Fig. 4c-ii). In contrast, papillary carcinoma samples sometimes show papillary growth of follicular cells exhibiting unique nuclear features including pale chromatin, nuclear membrane grooving, and pseudo-intranuclear inclusion (Fig. 4c-iii). Cystic carcinoma samples are difficult to image as the broken cyst tend to only create large voids in the sample histological images (Fig. 4c-iv**)**. In the case of tall-cell carcinoma (Fig. 4c-v), the majority of epithelial cells are elongated allowing for rapid progression throughout the thyroid and drastically increases the number of collagen fibers. Finally, the case of papillary carcinoma associated with Hashimoto thyroiditis shown in Fig. 4c-vi depicts a very staggered and heterogeneous structure in the sample but otherwise similar to usual papillary carcinoma.

As expected, compared to normal thyroid samples (0.06 ± 0.02 mN/mm), adenomatoid samples showed low stiffness (0.04 mN/mm) likely due to the uniform glands dominating the ROI analysis. Carcinomas with cystic change were identifiable through characteristically low stiffness and heterogeneity (0.02 ± 0.00 mN/mm) likely due to a distributed structure of fluid nodules and the reduction of transport vessels upon needle insertion into the cyst. Conversely, aggressive tall cell carcinoma showed significantly higher heterogeneity (6.30 ± 1.10 μN) due to increased fibrosis and calcification from rapid metastasis. A similar profile is shown for Hashimoto syndrome (5.75 ± 0.81 μN) in heterogeneity but can be differentiated from carcinoma using stiffness (Hashimoto 0.22 ± 0.02 mN/mm, Tall Cell 0.41 ± 0.03 mN/mm) due to reduced development of fibrosis while high heterogeneity from Hashimoto syndrome’s inhomogeneous cell distribution. Overall, despite several confounding factors including sex, age, development, and the state of health of their thyroids, STFN measurements of stiffness and stiffness heterogeneity readily identified malignant cases from benign cases based on either extremely high or low heterogeneity and stiffness (Fig. S5).

Moreover, the heterogeneity of healthy and adenomatoid samples was observed to differ by a factor of 2 from each of the malignant cases. Distinguishing between the variant types of papillary carcinoma also show similar ratios between each type but requires the auxiliary use of stiffness for some degenerate cases such as tall-cell and Hashimoto, where their heterogeneity profiles are similar. Using heterogeneity and stiffness in concert, the analysis yielded stiffness with differences by a factor of two thereby enabling biomechanics based identification between the two cases. Our study provides direct evidence for the diagnostic potential for fine needle force sensing approaches to obtain a quantitative biomechanical analysis of tissues. Our work provides direct evidence for the applicability of tissue biomechanics in assessing benign versus malignant thyroid lesions. More importantly, it also indicates the usefulness of biomechanical analysis in evaluating the aggressiveness of malignant lesions as a complementary approach for early diagnosis. STFN based stiffness and/or heterogeneity together reliably identified the different variants using heterogeneity (Fig. 4b) as validated by gold standard histological findings. However, a limitation of this study is that other variants of thyroid cancers, including the noninvasive follicular thyroid tumor with papillary like nuclei (NIFTP), follicular carcinoma, anaplastic carcinoma, medullary carcinoma, lymphoma remain to be evaluated.

## Conclusions and Outlook

Biomechanics plays an important role in normal and pathological tissue function. Tissue stiffness has been studied extensively within the field of cancer for diagnostic purposes using various imaging modalities. However, none of the currently available imaging modalities or biomechanical approach allows depth-independent yet direct quantitative assessment of biomechanical variations within the tissue microenvironments for early detection and management of thyroid cancers.

Our *ex vivo* thyroid study illustrates the capabilities of STFN as a mechano-profiling tool for tissue diagnosis. STFN provides an exclusive and novel approach to assess localized high resolution (cellular level) and quantitative biomechanical variations in thyroid tissues. The findings presented in the current *ex vivo* thyroid study, suggest the high potential for the STFN approach to be translated into an *in vivo* diagnostic device. Despite limited patient population size and need for further large clinical study, the results provide a solid foundation for further diagnostic assessment of quantitative tissue biomechanics *in vivo* to evaluate the risk of malignancy in thyroid lesions in a more extensive validation study in future. Even with the current small sample size we included a spectrum of lesions (to prove the concept) that there is substantial difference between benign versus malignant thyroid tissue. The technique can also be applied to lung, liver, pancreatic, or prostate cancer. This label-free technique enables complementary tumor tissue sampling required for genomic profiling and treatment planning, as needed. As a hand-held, low-cost technology, STFN could be ideal for diagnostic screening of solid tumors in medically underserved communities in the United States and low resource settings worldwide, with minimal or no access to pathology labs or experts.

## Supplementary information

Supplementary Information

## Data Availability

The data that support the findings of this study are available from the corresponding author, [SS, JR, JKG], upon reasonable request.
