# Supplementary material for: Piezoelectric needle sensor reveals mechanical heterogeneity in human thyroid tissue lesions
Source: Sci Rep. 2019 Jun 26;9:9282. doi: 10.1038/s41598-019-45730-x (PMC6594950; doi:10.1038/s41598-019-45730-x)
Supplement: Supplementary file 1 — Supplementary Information [file 41598_2019_45730_MOESM1_ESM.pdf]

SUPPLEMENTARY INFORMATION

Piezoelectric needle sensor reveals mechanical heterogeneity in human thyroid tissue lesions

Shivani Sharma[1,2,3,4] *⊥,Renato Aguilera[5]⊥, JianYu Rao[1,2,3,4]*, & James K. Gimzewski[3,5]*
[1]Department of Pathology and Laboratory Medicine, David Geffen School of Medicine at UCLA, Los Angeles, CA
[2]California NanoSystems Institute, Los Angeles, CA
[3]Jonsson Comprehensive Cancer Center, Los Angeles, CA
[4]Clinical and Translational Science Institute, University of California Los Angeles, CA
[5]Department of Chemistry and Biochemistry, University of California Los Angeles, CA

⊥ Co-first authors (S Sharma, R Aguilera)

Supplemental

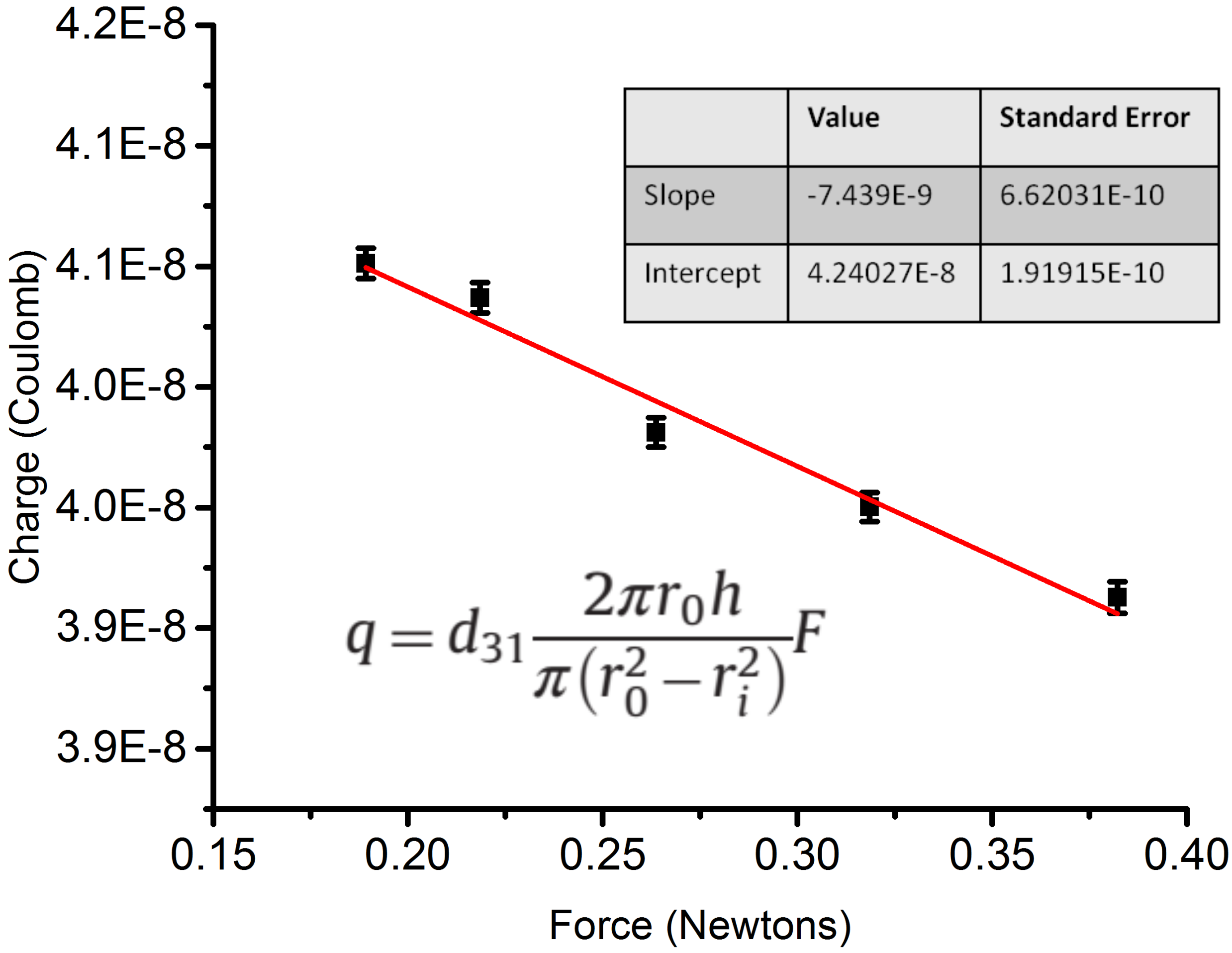

Supplemental Figure 1. Piezoelectric Coefficient Calibration. Calibration of the piezoelectric element was performed using standardized weights loaded on a custom built apparatus to determine the d_{31} coefficient. A statistical number of measurements were taken and a least square linear regression algorithm was used to determine the d_{31} (-81.2 x 10^{-12} m / V) for the device using r_0 (outer radius, 0.0625 in.), r_i (inner radius, 0.0425 in.) and h (height, 0.495 in.) using the above formula.

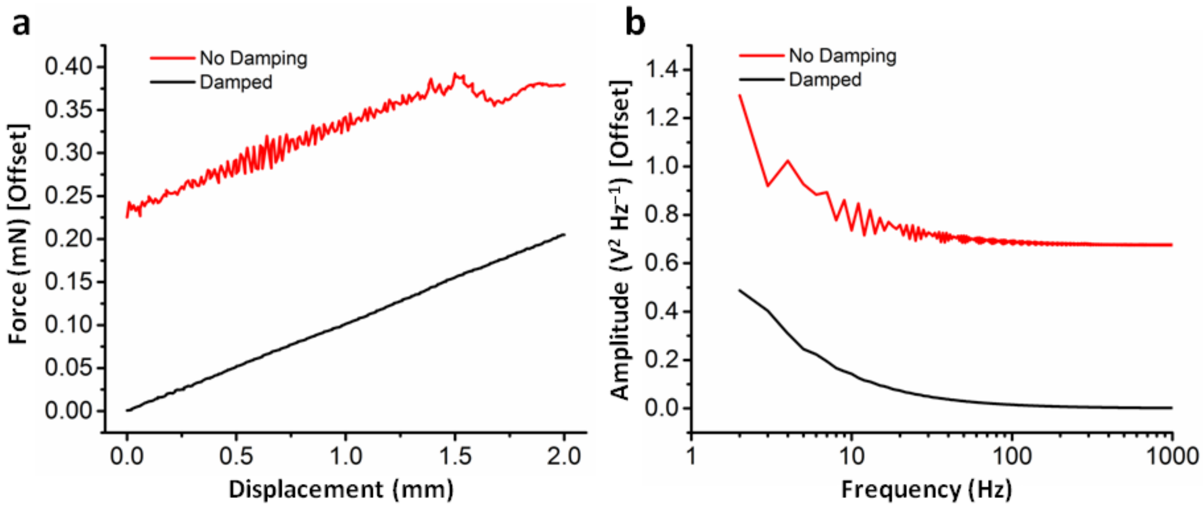

Supplemental Figure 2. Vibrational Liquid Filter Performance. The frequency response of the STFN device was measured with (black) and without (red) the vibrational liquid filter. The STFN was used on a gel sample showing the force curves (**a**) and the Fourier Transform in (**b**). Peak-to-peak analysis between the damped and non-damped apparatus from (**b**) show a reduction of ~3 dB in noise amplitude.

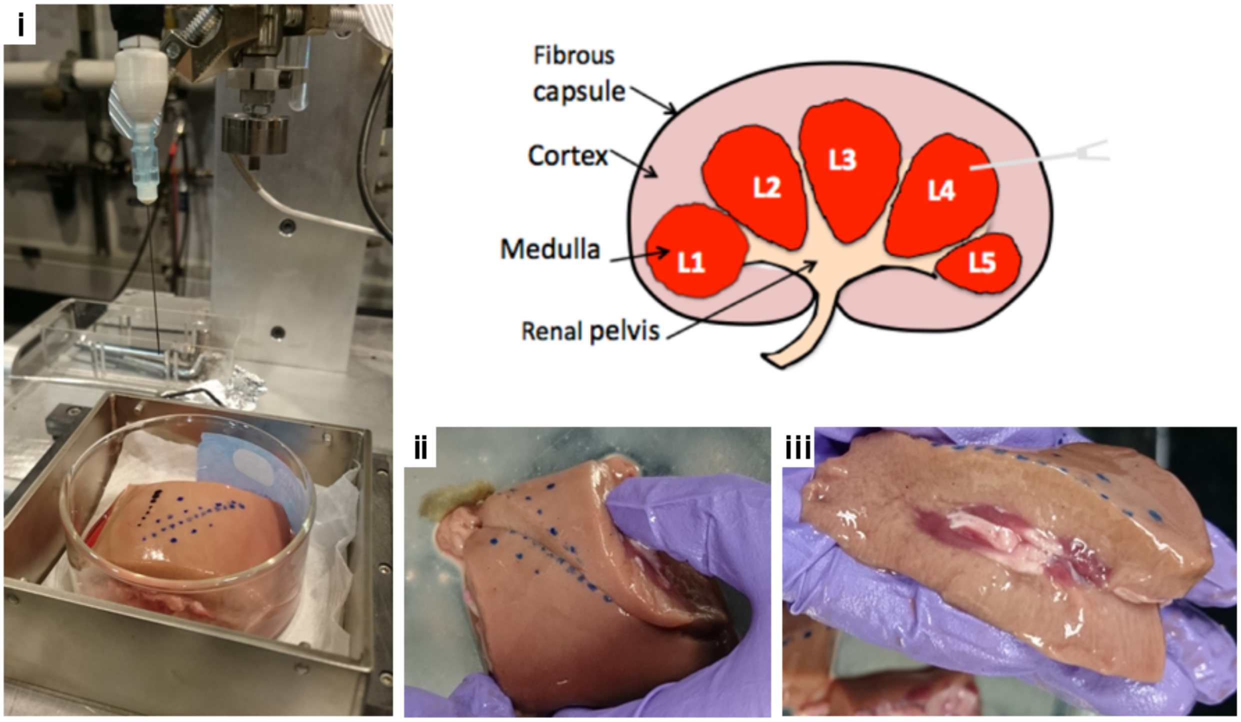

Supplemental Figure 3. Porcine Kidney Dissection and Analysis. A post-measurement dissection of the porcine kidney sample was conducted to elucidate the mechanical profile of the porcine kidney. Measured areas are marked with blue ink (**i**) and conducted in ascending order. Dissection normal to the penetration (**ii**) reveals a hard renal structure (**iii**) near the incident of penetration and exit**.**

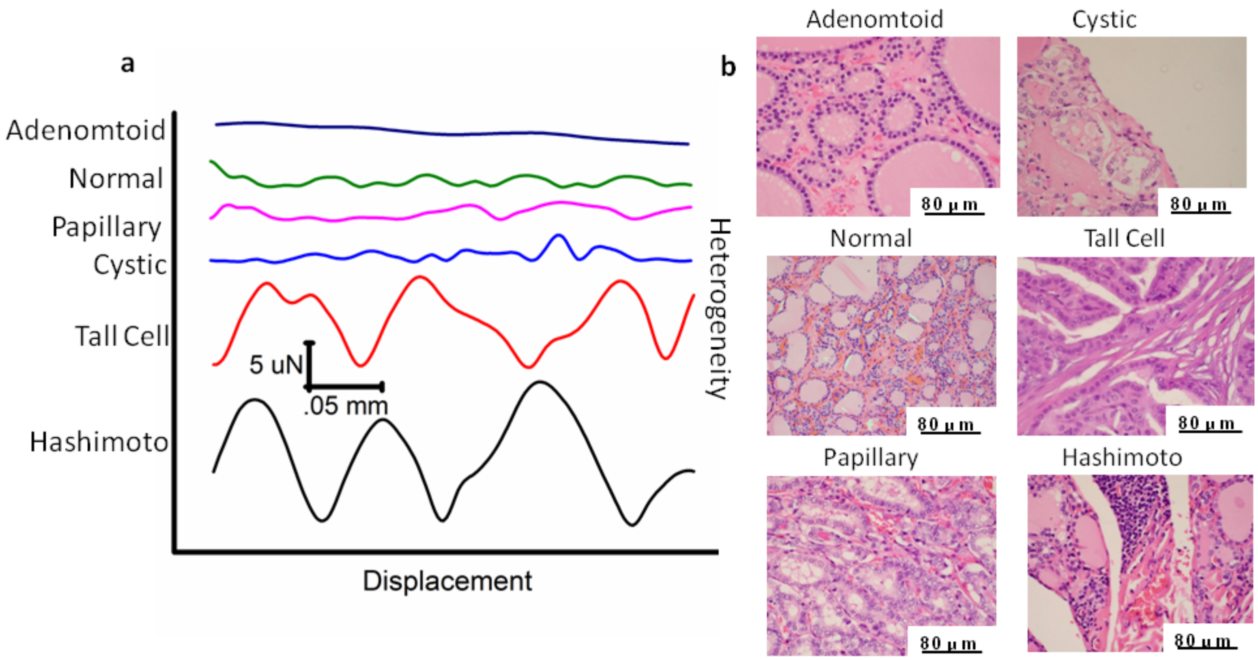

Supplemental Figure 4. Heterogeneity Increase due to Fibrosis/Calcifications. Ex vivo STFN measurements of human thyroids showing the distribution of tissue heterogeneity responses **a** observed for thyroid carcinoma and healthy thyroids. Increased tissue stiffness heterogeneity observed for malignant thyroid samples compared to non-tumor thyroid samples, corresponds well with malignant tissue histology, shown in **b** displaying increased stroma density shows histology for a non-tumor sample with little intervening stroma for comparison.

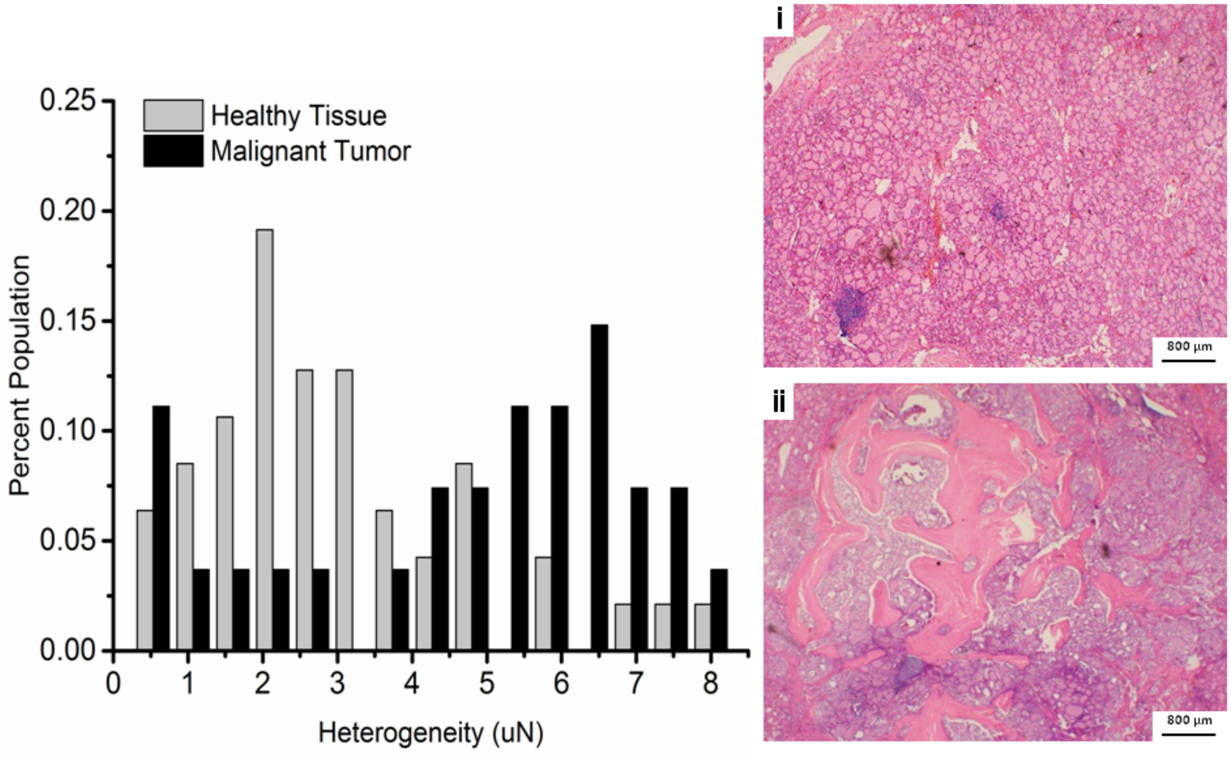

Supplemental Figure 5. Varied STFN Responses of Thyroid Carcinoma and Healthy Thyroid. Representative ex vivo measurements of human thyroid show clear distinctive response between malignant and benign samples. Benign measurements from adenomtoid and normal (healthy) thyroid depict low heterogeneity within the tissue **i**, where the heterogeneity is measured at a random location on the tissue. Measurements on malignant samples (Papillary to Hashimoto) depict high heterogeneity likely due to calcification around the nodule **ii**.
